# Extracorporeal Shockwave Therapy Treatment in Upper Limb Diseases: A Systematic Review

**DOI:** 10.3390/jcm9020453

**Published:** 2020-02-06

**Authors:** Gianluca Testa, Andrea Vescio, Stefano Perez, Alberto Consoli, Luciano Costarella, Giuseppe Sessa, Vito Pavone

**Affiliations:** Department of General Surgery and Medical Surgical Specialties, Section of Orthopedics and Traumatology, A.O.U. Policlinico-Vittorio Emanuele, University of Catania, 95123 Catania, Italy; gianpavel@hotmail.com (G.T.); stefanoperez91@gmail.com (S.P.); albeconsoli@gmail.com (A.C.); lcostarella@yahoo.it (L.C.); giusessa@unict.it (G.S.); vitopavone@hotmail.com (V.P.)

**Keywords:** upper limb soft tissue diseases, conservative treatment, extracorporeal shockwave therapy, chronic tendinopathies

## Abstract

Background: Rotator cuff tendinopathy (RCT), subacromial impingement (SAIS), and medial (MEP) and lateral (LEP) epicondylitis are the most common causes of upper limb pain caused by microtrauma and degeneration. There are several therapeutic choices to manage these disorders: extracorporeal shockwave therapy (ESWT) has become a valuable option. Methods: A systematic review of two electronic medical databases was performed by two independent authors, using the following inclusion criteria: RCT, SAIS, MEP, and LEP, ESWT therapy without surgical treatment, with symptoms duration more than 2 months, and at least 6 months of follow-up. Studies of any level of evidence, reporting clinical results, and dealing with ESWT therapy and RCT, SAIS, MEP, and LEP were included. Results: A total of 822 articles were found. At the end of the first screening, following the previously described selection criteria, we selected 186 articles eligible for full-text reading. Ultimately, after full-text reading, and reference list check, we selected 26 articles following previously written criteria. Conclusions: ESWT is a safe and effective treatment of soft tissue diseases of the upper limbs. Even in the minority cases when unsatisfied results were recorded, high energy shockwaves were nevertheless suggested in prevision of surgical treatment.

## 1. Introduction

Rotator cuff tendinopathy (RCT), subacromial impingement (SAIS), and medial (MEP) and lateral (LEP) epicondylitis are the most common causes of upper limb pain [1]. Calcific tendinitis is a painful disorder characterized by either single or multiple presences of calcium deposits in the tendon or subacromial bursa [2], while the term noncalcific tendinitis refers to tendinitis without calcium deposits [1]. Both conditions are common in the shoulder and elbow and affect both sedentary people and athletes [3].

RCT is the most common source of shoulder pain, and its prevalence is estimated to be 2%–3.8% in the general population [4]. Subacromial impingement syndrome (SAIS) is a pathological state of the rotator cuff tendons [5], resulting from mechanical impingement causing 50%–70% of shoulder pain cases [6]. MEP, or “golfer’s elbow”, is the result of common flexor tendon (CFT) microtrauma and degeneration, and might affect <1% of the general population and as many as 3.8% to 8.2% of patients in occupational settings, and typically occurs from the fourth decade of life [7]. Lateral epicondylitis (LEP) is a common chronic inflammatory degeneration of the wrist extensor tendons at their insertion to the lateral epicondyle of the humerus, affecting 1%–3% of the general population, especially between the ages of 30 and 70, without gender predisposition [8]. Inflammation and chronic degeneration are the key conditions in these pathogeneses, and their natural history is influenced by the quality of the tendon tissue, along with mechanical overuse or misuse [3]. The risk of recurrence is very high, and no definite therapy is available [9]. Numerous therapeutic choices (surgical and conservative) can be used to manage these kinds of disorders. However, the goals remain similar: to relieve acute symptomatology, rehabilitate the pathologic tendon, and prevent future recurrence. As with many chronic tendinopathies, nonsurgical therapy is the first choice for treatment [9]. Standard management regimens of these diseases include rest, cryotherapy, activity modification, stretching exercises, nonsteroidal anti-inflammatory drugs (NSAIDs), and eccentric loading [10].

Extracorporeal shockwave therapy (ESWT), initially introduced for the treatment of nephrolithiasis, has become a viable option for the treatment of various soft-tissue disorders [11], especially the chronic Achilles tendinopathy [12,13] and plantar fasciitis [14], as well as bone defects [15]. Although the mechanism by which ESWT produces a therapeutic effect is not completely understood [16], Wang and colleagues supposed that the repeated microtrauma could stimulate the ingrowth of neovascularization associated with the up-regulation of angiogenic and osteogenic growth factors [17]. Similarly, the calcium reabsorption is not clear, the principal hypothesis is mechanical, suggesting the fragmentation of the calcium deposit is caused by the pressure wave directly on the site [18]; on the other hand, some authors have speculated a cavitation effect, due to the ESWT: in fact, the highly intensive shockwaves should first affect the integrity of the calcifications and, subsequently, destroy the deposit [19]. The molecular hypothesis advances the active role of the inflammatory response and leukocyte chemotaxis to generate the phagocytosis of the calcium deposit [16]. Moreover, ESWT may reduce pain through hyperstimulation of nociceptors/gate-control theory of pain transmission, altered pain receptor neurotransmission, and by increasing local pain inhibiting substances [20]. Stimulation of nociceptive C-fibers may not only play a role in analgesia, but also in tendon remodeling, as it may increase the release of neuropeptides, causing fibroblast stimulation and vasodilation [20]. According to energy levels the ESWT can be distinguished in: (1) low-energy (flux density (FD) of up to 0.08 mJ/mm^2^); (2) moderate (FD of between 0.09 and 0.28 mJ/mm^2^,); and (3) high energy (0.6 mJ/mm^2^) shock waves [21].

Many studies have attempted to investigate the efficacy of ESWT on the acute and chronic tendinopathy of the upper limb, but orthopedic surgeons have yet to reach consensus on this. The purpose of this systematic review is to analyze the available literature, providing an update on the evidence related to the upper limb tendinopathy and subacromial impingement syndrome patients treated with ESWT.

## 2. Experimental Section

### 2.1. Study Selection

According to the guidelines of the Preferred Reporting Items for Systematic Reviews and Meta-Analyses (PRISMA) [22], PubMed and Science Direct databases were systematically reviewed by two independent authors (AV and SP). The research string used was “(Cuff rotator tendinopathy OR Lateral Epicondylitis tendinopathy OR Tennis Elbow OR Lateral Epicondylitis tendinopathy OR Golfer’s Elbow OR Subacromial impingement syndrome) NOT (Cuff Rotator rupture OR Cuff Rotator lesion OR Fracture) AND (ESWT OR Extracorporeal Shockwave Therapy)”. From each included original article, a standard data entry form was utilized to extract the number of patients, mean age at treatment, sex, type of treatment, minimum time of symptoms, duration of treatment, complication rate and complication type, follow-up, and year of the study. 

Study quality assessment was performed in duplicate by two independent reviewers (AV and GT). Discussing conflicts about data were resolved by consultation with a senior surgeon (VP). 

### 2.2. Inclusion and Exclusion Criteria

Eligible studies for the present systematic review included extracorporeal shockwave therapy treatment in the upper limb. The initial titles and abstracts screening was made using the following inclusion criteria: Rotator cuff tendinopathy (RCT), subacromial impingement (SAIS), and medial (MEP) and lateral (LEP) epicondylitis, ESWT therapy without operative treatment, with more of 2 months symptoms and a minimum average of 6-months follow-up. 

The exclusion criteria were groups of patients with primary or secondary surgical treatment, symptoms for less of 6 months, and animal trials. All remaining duplicates, articles focused on other topics or with poor scientific methodology and accessible abstract were excluded. 

### 2.3. Risk of Bias Assessment

In this systematic review, risk of bias assessment was performed according to the ROBINS-I tool for non-randomized studies [23], consisting of three-stage assessment of the studies included. The first stage regards the planning of the systematic review, the second stage is the assessment of the common bias possibly found in these studies, and the latter is about the overall risk of bias. 

Three authors (AV, AC, and GT) performed the evaluation independently. Any discrepancy was discussed with the senior investigator (VP) for the final decision. All the raters agreed on the final result of every stage of the assessment (Table 1 and Table 2).

## 3. Results

### 3.1. Included Studies

**Figure 1:**
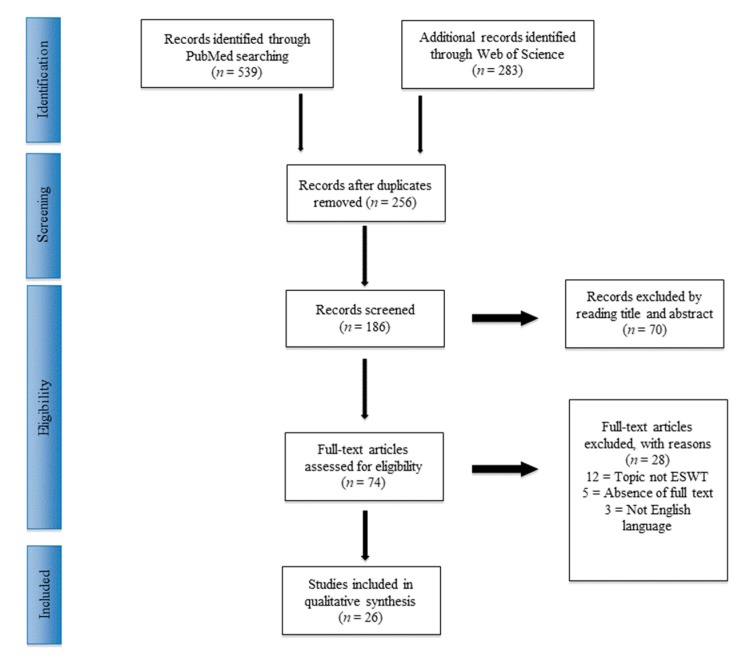
PRISMA (Preferred Reporting Items for Systematic Reviews and Meta-Analysis) flowchart of the systematic literature review.

A total of 822 articles were found. After the exclusion of duplicates, 186 articles were selected. At the end of the first screening, following the previously described selection criteria, we selected 74 articles eligible for full-text reading. Metanalysis or systematic reviews were excluded from the study. Ultimately, after full-text reading, and checking of the reference list, we selected 26 articles, composed of randomized controlled human trials (hRCT) and prospective and retrospective cohort or series studies, following previously written criteria. A PRISMA [21] flowchart of the method of selection and screening is provided (Figure 1). The main findings of the included articles were summarized (Table 1 and Table 2). 

### 3.2. Shoulder

All of the articles [11,24,25,26,27,28,29,30,31,32,33,34,35,36,37,38] included in the study focus on calcific tendinopathy of the shoulder, non-calcific tendinopathy of the shoulder, and subacromial impingement syndrome (SAIS).

#### 3.2.1. Calcific Tendinopathy of the Rotator Cuff (CTRC)

Many articles [24,25] demonstrated a decrease of the pain and the amount of calcification [26,27,28] in patients treated with ESWT in calcific tendinopathy of the shoulder. Also, ESWT and ESWT associated with kinesio taping (KT) groups, Frassanito et al. [29] reported better outcome and a faster recovery in patients treated with functional taping. The combination of ESWT and dietary supplement (DS) containing methylsulfonylmethane, hydrolyzed swine collagen (Type I and Type II), l-arginine and l-lysine, vitamin C, condroitin sulfate, glucosamine, and curcuma longa has been shown to provide a greater and faster pain relief, with a significant reduction of NSAIDs consumption [11]. Chou et al. [30] emphasized that patients with Gartner and Heyer type I calcification, calcification >15 mm, and the duration of symptoms >11 months had poorer outcome after ESWT. 

#### 3.2.2. Non-Calcific Tendinopathy of the Rotator Cuff (NTRC)

Regarding the treatment of non-calcific tendinopathy of the rotator cuff, the evidence supporting the ESWT treatment in short- and long-term is controversial. Li et al. [31] found that, considering a total of 84 patients, pain symptomatology was improved in the high dose ESWT group. Galasso et al. [32] detected a remarkable short-term efficacy in functional recovery in ESWT patients relative to a placebo group. Wu et al. indicated that the high-dose ESWT posed superior clinical efficacy in type II/III calcification tendinosis compared to type I calcification and noncalcific shoulder tendinosis [33]. In non-calcific supraspinatus tendinopathy patients treated with ESWT, Efe et al. [34] did not record any effect on function or pain improvement after 10 years. Speed et al. [35] reported a significant and sustained placebo effect after moderate doses of ESWT, but no evidence of added benefit when compared with the sham treatment.

#### 3.2.3. Subacromial Impingement Syndrome (SAIS)

As reported by Circi et al. [36], ESWT has been considered effective in the treatment of impingement syndrome in the early period, both for pain and functional outcome, regardless of acromion morphology. The study of Kvalvaag et al. [37], which included 143 subjects, reported no differences between the ESWT group and the control. In SAIS patients, superior functional recovery, muscle endurance, and decrease of pain in the short to medium term were recorded when ESWT was associated with isokinetic exercises [38]. 

### 3.3. Elbow

Thirteen studies assessed the outcome of patients treated with ESWT compared to a control group, tenotomy, acupuncture, local infiltration of corticosteroids, or cryoultrasound in medial and lateral epicondylitis.

#### 3.3.1. Lateral Epicondylitis (LEP)

The results of the studies investing ESWT treatment of lateral epicondylitis affected patients are controversial. Similar to other authors [39,40,41], Pettrone et al. [42], in a study involving 114 patients, obtained satisfying results in the management of epicondylitis with ESWT (vs. a placebo group). When an ultrasonography-guided [43] approach was used, the authors reported a positive response in 75.7% of the patients after the first treatment. On the contrary, other authors [35,44] reported a similar outcome relative to the placebo group. Compared to surgical treatment, such as tenotomy, ESWT represented a valid alternative after long-term follow up [45], as well as compared to the local infiltration of corticosteroids [46]. Vulpiani et al. [47] highlighted an improvement of functional recovery in chronic epicondylitis in patients treated with ESWT relative to the cryoultrasound group after 12 months. 

#### 3.3.2. Medial Epicondylitis (MEP)

Lee et al. [46] compared the ESWT treatment of medial epicondylitis in the acute phase with local infiltration of corticosteroids. Although the corticosteroids injections had more satisfying results in the short term (after 1 year), the ESWT patients had a better functional outcome. Similar short-term values were achieved in ESWT patients compared to acupuncture treatment [48].

## 4. Discussion

### 4.1. General Consideration

The findings addressing the treatment with ESWT in the chronic tendinopathies and diseases of the upper limbs are somewhat heterogeneous; many controversies remain unresolved. There is no consensus about the number of sessions of ESWT required. Generally, the protocols provide between one and six sessions per week, increasing the number of sessions does not seem to improve outcomes. There is no consensus regarding the energy setting that should be used [25]. The shock wave generator, the number of impulses, the focusing of the shockwave concerning the tendon insertion, the number and the interval between each treatment session, different stages of the disease, and types of calcification all are important factors that must be carefully considered [25,27,49]. 

ESWT requires expensive shockwave delivery apparatus and several clinical sessions [30].

Contraindications for ESWT included pregnancy, acute infection, malignant tumor and coagulopathy, fracture or calcific tendinitis coexisting with a rotator cuff tear [30].

Common complications and advised effects after ESWT include transient pain, skin erythema, pain, and local swelling [20].

At the best knowledge of the authors, this is the first article analyzing and summarizing the main literature evidences of the functional outcomes in patients affected by shoulder and elbow soft tissue diseases and treated with ESW.

### 4.2. Shoulder

Several [25,26,27,28,29,30,31,32,33,34] studies reported satisfying results for functional outcome and decrease of pain after ESWT treatment in CTRC, NTRC, and SAIS affected patients. Some authors even reported the use of ESWT to be more effective than the use of transcutaneous electric nerve stimulation (TENS) [26] in patients with chronic calcific tendinitis of the shoulder, especially of the rotator cuff with arc-type calcific plaque [28]. Galasso [32] and Li et al. [31], despite Speed et al. [35] showed the contrary, reporting encouraging effect for treated patients with CTRC and NTRC in the short-term follow-up, unconfirming improvement in long-term compared to the placebo group [34]. Several causes could influence the outcome at 10 years: (1) other treatment interventions, in particular, physical therapy with or without a focus on scapular kinematics; (2) change in occupation and workload; (3) changes in lifestyle; and (4) changes in sports activity.

Chou et al. [30] aimed to predict the outcome in these subjects, identifying Gartner and Heyer type I classification (calcification >15 mm, and duration of symptoms >11 months) as negative prognostic parameters [33]. The timing could be considered as an additional predictor factor; in fact, Malliaropoulos et al. [24] found a correlation between pre-treatment and post-treatment pain duration, suggesting to start the management as soon as possible. 

The application of taping as adjuvant therapy is interesting; in fact, KT seems to reinforce the analgesic and regenerative action of ESWT in the short term and promoted a faster therapeutic response at the mid-long term. Some authors [29] have speculated that KT performs its therapeutic functions mainly by proprioceptive feedback, through an immediate and constant stimulation of the mechanoreceptors in the skin, protecting the joint and reducing wrong movements. KT is advantageous because it is non-invasive, non-pharmacological, localized, and relatively cheap. On the other hand, further research is necessary to define the therapeutic indications, identify the best application methods, and clarify which factors determine the clinical result. Similarly, nutraceutical supplementation seems to improve the function and pain symptomatology in CTRC and NTRC patients [11]. 

Regarding ESWT treatment of SAIS, Circi et al. [36] reported successful results in a manner that is independent of acromion morphology in recovering the pain and functional outcome. Santamato et al. [38] encouraged the association between the ESWT and isokinetic exercise, explaining how the pathogenesis of this syndrome is multifactorial, resulting by the combination of several factors, including degenerative process, the alerted kinematics, postural aberrations, muscle deficit of performance [38]. Nonetheless, in a randomized, double-blind trial based on a large sample, Kvalvaag et al. [37], contradicted the efficiency of ESWT in subacromial impingement relative to patients treated with physical therapy.

### 4.3. Elbow

More than 40 different modalities of treatment of medial and lateral epicondylitis, used either alone or in combination, have been reported [50]. Numerous studies have reported good results of ESWT in decreasing of pain, improving the functional outcome and grip strength, in calcific and non-calcific tendinopathies [39] with a chronic presentation. Moreover, Köksa et al. [41] described an improvement of symptomatology and functionality, suggesting not only the absence of exacerbation or increase of the inflammation, but recommending ESWT for acute symptoms, thereby avoiding progression to the chronic phase or suffering from long-term pain.

Nevertheless, the efficacy of ESWT was not superior to other treatments. In fact, Lee et al. [46] reported that local steroid injection was more effective at the beginning, and similar results were reported after the first 2 weeks of therapy when the shockwaves were compared to acupuncture [47]. 

Some authors, who have described unsatisfied results in the use of low energy ESWT, have nevertheless suggested applying a method involving alternative doses and/or different dosage intervals [30] or before surgical treatment in refractory or relapsing cases [43,44].

Several adjuvants therapies or methods have been described to improve the effectiveness of ESWT. The use of focal ultrasonography improved the functionality of shockwaves [43], as well as the use of DS, because of their advantage to be not pharmacologic and to contain compounds with modulatory effects on inflammation [3]. The combination of ESWT and DS produce an increased bioavailability of the supplement to the tendon tissue, due to the neo-angiogenic properties [51,52], which results in a decrease in the use of NSAIDs [11].

### 4.4. Limits of the Study

The heterogenous of the scores considered to assess the patient functional outcome and the absence of ESWT standard protocol are the main limits in the comparison of studies results. We extensively searched and identified all relevant ESWT in upper limb soft tissue diseases articles. Therefore, risk of bias assessment showed moderate overall risk which could influence our analysis.

## 5. Conclusions

Extracorporeal shock wave therapy is a safe and effective treatment of upper limbs soft tissue diseases. Despite similar result to other therapies in short and middle terms, numerous studies have suggested the use of high energy shockwaves in chronic tendinopathies. In the minority of cases when unsatisfied results were recorded, high energy shockwaves were nevertheless suggested in prevision of surgical treatment. Adjuvant therapies, as the kinesio taping and dietary supplementation, seems to be useful in the treatment of upper limb soft tissues diseases but further findings are mandatory, we encourage high-profile clinical studies to investigate adjuvant therapy or other methods able to improve the effectiveness of ESWT. The literature available on the ESWT treatment in upper limb soft tissue diseases presents major limitations in terms of great heterogeneity and lack of high-profile studies. Further randomized control trials are strongly encouraged.

## Figures and Tables

**Table 1:** The main findings of shoulder tendinopathies included case-control and cohort studies.

Ref	Authors	No. of Patients	Group	No. of Impulses (Time a Week)	Follow-Up	Results Summery
[11]	Vitali et al.	30	ESWT vs ESWT + DS	1700–2000 (1)	0, 7, 30 and 60 days	*At 2 months follow-up, ESWT+DS group had significant improvement of functional outcome (p value = 0.0002) and pain compared to the only ESWT group. A significant reduction of painkiller was in the ESWT+DS group after one month (p value = 0.0308) and after 2 months (p value = 0.0061).* (ESWT+DS (UCLA score = 27 (26–29); VAS = 2 (1–3); NSAIDs = 0%); (ESWT (UCLA score 23(17–25); VAS=5(4–7); NSAIDs = 40%)
[24]	Malliaropoulos et al.	67	rESWT	1500 (>3)	0, 1, 3, 12 months	*The study reported the 92% of satisfied patients after 1 year of treatment, 52% of pain decreased after the first treatment, 62% after 30 days, 75% at 3 months and 88% at 1 year follow up. Recurrence rate of the 7%.* VAS (baseline = 6.7 ± 1.1; Post-treatment=3.2 ± 0.8; 1st month=2.6 ± 0.9; 3rd month=1.7 ± 1.0; 1 year = 0.8 ± 1.0)
[25]	Cosentino et al.	70	ESWT vs placebo	1200 (3)	0, after the treatment, 1 and 6 months	*The pain (p-value < 0.05)was significantly reduced compared to the baseline and improved the functional outcome (p-value < 0.05). Calcium complete reabsorption in the thirty-one to forty per-cent of the patients, and partial in 40% of cases.* (ESWT CS( baseline = 45 points (pain 5.2, activities of daily living 9.6, range of motion 23.2, power 7); final follow-up = 71 points (pain 9.8,; activities of daily living 13; range of motion 32; power 16.2))(Placebo Constant Score (baseline = 48 points, final follow-up = 50 points).
[26]	Del Castillo-González et al.	243	ESWT vs UGPL	2000 (2)	0, 3, 6, 12 months	*Sixty-five per-cent of pain free patient, 55.6% after 1 year. * (VAS (baseline=7.43 ± 0.99; 1 year 2.0 ± 0.4)
[27]	Gerdesmeyer et al.	144	High ESWT vs low ESWT vs placebo	1500 (1)	0, 3, 6, 12 months	*High ESWT and low ESWT group recorded the a significant improvement compared to placebo group and baseline. High energy group results were superior the low-energy group results. * (CS at 6-month follow-up (High ESWT=31.0 (26.7–35.3); low ESWT = 15.0 (10.2–19.8); placebo = 6.6(1.4–11.8)
[28]	Pan et al.	63	ESWT vs TENS	2000 (Nd)	0, 2, 4, 12 weeks	*Significant improvement of the shoulder joint ROM, activities day-livings, and decrease of the pain condition.* (ESWT = CS(baseline = 63.77 ± 14.22; final difference =+28.31 ± 13.10); VAS(baseline=6.50±1.81; final difference = -4.08±2.59)); (TENS=CS(baseline=65.66 ± 15.84; final difference = + 11.86 ± 13.32); VAS(baseline = 6.70 ± 1.42; final difference = −1.74 ± 2.20)
[29]	Frassinito et al.	42	ESWT vs ESWT + KT	1800 (3)	0, 1, 4, 12 weeks	*At the one-week follow up, the authors recorded a significant reduction in pain and functional enhancement.* Baseline(VAS = ESWT+KT =6. 6±1.5(6.0,7.2); ESWT = 6.7 ± 1.1(6.2,7.1) DASH(ESWT+KT = 33.6 ± 12.1(28.4,38.8); ESWT = 31.1 ± 8.9 (27.3, 34.9)); Final difference(ESWT+KT VAS =−5.4; ESWT VAS = −4.3; ESWT+KT DASH = −25.9; ESWT DASH = −17.4)
[30]	Chou et al.	241	ESWT	3000 (1−2)	0, 3, 6, 12 months	*After ESWT, improvement of outcome and pain. After the first treatment, 90.5% of complete calcium deposit fragmentation and resorption and the remaining 9.5% after the second. Odds ratio Type I calcification of 24.8 than the other types. Additional one month of symptoms each 1 mm increase of calcific deposit.* CS (Baseline = 53.7 ± 10.2(24−37); After treatment 90.0 ± 16.4(33−100))
[31]	Li et al.	84	ESWT vs placebo	3000 (2)	0, 4, 8 weeks	*At the 4(p < 0.05) and 8(p < 0.01) weeks follow-up, significant improvement of pain and functional outcome.* CS (Baseline(ESWT = 53.7 ± 14.1; 56.2 ± 14.4) 4-weeks difference(ESWT = 19.4(10.1, 28.5); placebo = 10.3(5.4,17.7)); 8-weeks difference (ESWT = 27.2(18.6, 38.3); placebo = 14.1 (8.8, 20.2))
[32]	Galasso et al.	20	ESWT vs placebo	3000 (1)	0, 6, 12 weeks and 3 months	*Satisfied patients in ESWT group 63.7% and 22.3% of control group. Improvement of pain and functional outcome after shockwaves treatment compare to the other cohort.* CS Baseline (ESWT = 42.45 ± 9.83 (29–61) placebo = 41.67 ± 12.53 (20–57)); CS 6 weeks ESWT = 64 ± 16.6 (32–87); placebo = 43.11 ± 19.16 (18–70); CS 3 months ESWT = 74.09 ± 20.56 (39–98) placebo = 48 ± 22.3 (17–79).
[33]	Wu et al.	20	ESWT	3000 (1)	0, 3, 6, 12 months	*At one year follow up, decreasing of pain symptomatology and increasing of the shoulder functionality in chronic shoulder tendinosis and type II and type III calcification patients. Similar results in type I calcified and noncalcified tendinitis patients. * CS Baseline (No and I Calcified CS = 52.5 ± 14.5(21–74) Calcified CS=49.7 ± 9.03(33–62)); After treatment (No and I Calcified CS = 78.7 ± 18.3(38–98); Calcified CS=71.1 ± 17.8(44–98)
[34]	Efe et al.	29	ESWT vs placebo	3000 (Nd)	0 and 10 years	*At 10 years follow-up, increase of functional outcome and decrease of pain differences compare to the baseline in ESWT group but not significant differences compared to placebo group. Eight surgical treated patients between 1- and 10-year follow-ups. * The CS at last follow-up placebo = 99 ± 31; ESWT = 105 ± 24) Differences between baseline and 10-year follow-up data were significant (*p* = 0.02)
[35]	Speed et al.	74	ESWT vs placebo	1500 (Nd)	0, 1 and 3 months	* The 50% improvement of functional score was recorded in the thirty-five per-cent of ESWT group and the 45% of the placebo subjects after 90 days. Significant decrease of pain in both groups. At 6-month follow-up, similar results in the ESWT and placebo cohort.* ESWT( baseline = 53.6 ± 20.2(13 to 89); 1 month = 48.7 ± 21.0 (7 to 83); 2 month 46.1 ± 22.4 (9 to 88) 3 month 34.7 ± 26.6 (2 to 90); 6 month = 24.1 ± 22.9 (0 to 82)); placebo( baseline = 59.5 ± 16.1 (16 to 90); 1 month = 58.5 ± 19.7 (13 to 93); 2 month = 48.6 ± 23.8 (3 to 90); 2 month = 39.7 ± 27.7 (5 to 96); 6 month = 34.9 ± 31.7 (0 to 95))
[36]	Circi et al.	30	ESWT and Acromion type	1500 (3)	0 and post-treatment	*Improvement of functional score and pain in all patients, no differences between acromion types subgroups. * Acromion type 1 = SPADI(baseline = 47.9 ± 22.4 (16–90);post-treatment = 33.0 ± 19.2(2–60); Acromion type 2 = SPADI(baseline= 57.5 ± 26.3 (14–95); post-treatment = 39.5 ± 24.6 (9–70)); Acromion type 3 = SPADI(baseline = 59.6 ± 27.9 (26–91); post-treatment = 43.6 ± 23.4)
[37]	Kvalvaag et al.	143	rESWT + FKT vs placebo +FKT	2000 (4)	0 and 1 year	*Successful result in the 51.4% of ESWT+FKT group and the 53.6% of placebo + FKT group after 12 months. No significant differences between cohorts. * rESWT+FKT=SPADI(baseline = 51.9 ± 16.7;post-treatment = 28.3 ± 19.2(2–60); placebo + FKT = SPADI(baseline = 51.8 ± 17.5; post-treatment = 26.9 ± 27.3);
[38]	Santamato et al.	30	ESWT vs ESWT + FKT	700 (3)	0, 10 days and 2 months	*Improvement of pain and shoulder functionality in both groups compared to baseline. Better results in ESWT+FKT cohort than ESWT-only group at 10- and 60-days follow-up. * Group ESWT CS (Baseline = 49.7 ± 7.9(45.4, 54.1); 10days = 65.1 ± 7.7(60.8, 69.3); 2-month= 75.9 ± 6.7 (72.2, 79.6)) Group ESWT + FKT(Baseline = 45.6 ± 9.8 (40.2, 51.0); 10days = 63.6 ± 8.7 (58.8, 68.4); 2-month = 92.1 ± 6.3 (88.6, 95.6))

ESWT = extracorporeal shockwave therapy; rESWT = radial extracorporeal shockwave therapy; DS = dietary supplement; UGPL = ultrasound-guided percutaneous lavage; TENS = transcutaneous electric nerve stimulation; FKT = physical therapy; KT = kinesio taping; UCLA score = University of California Los Angeles shoulder score; VAS = Visual Analogue scale; CS = constant score; DASH = disabilities of the arm, shoulder and hand questionnaire; SPADI = Shoulder Pain And Disability Index; NSAIDs = nonsteroidal anti-inflammatory drug.

**Table 2:** The main findings of elbow tendinopathies included case-control and cohort studies.

Ref	Authors	No. of Patients	Group	No. of Impulses (Time a Week)	Follow-Up	Results Summery
[11]	Vitali et al.	30	ESWT vs ESWT + DS	1700-2000 (1)	0, 7, 30 and 60 days	*In the ESWT, + DS patients were recorded an early significant functional betterment at 7-, 30-, and 60-days follow-up compared to the other group. Reduction of painkiller in 1 and 2 month s(p value = 0.0001 and p value = 0.0053, respectively)*. (ESWT + DS (Mayo = 27 (26–29); VAS = 2 (1–3); NSAIDs = 7%);
[39]	Park et al.	43	ESWT	2000 (1)	0, 3 and 6 months	*Improvement of functional score and pain in all patients*. (100-point score (F_(df = 1)_ = 97.801, *p* < 0.001) and Nirschl Pain Phase scaleF_(df = 1)_ = 63.061, *p* < 0.0001)
[40]	Bayram et al.	12	ESWT	2000 (3)	0 and 1 month	*Decrease of pain at the rest, compression and activities 1-month after the treatment compare to baseline (p < 0.05). Patient’s and physician’s global post treatment self assessment scores were improved comparing the values pre- and post-operatively (p < 0.05).* (PRTEE pret = 91.50 ± 11.24; PRTEE postt 1. month 55.83 ± 11.69)
[41]	Koskal et al.	54	ESWT	2000 (2)	0, 2, 12, and 24 weeks	*In each evaluation, improvement of pain symptomatology while resting, stretching, working, and nighttime pain compared to the baseline.* Pretherapy(VAS resting = 0.79 ± 1.91; VAS stretching = 7.25 ± 1.29; VAS pressed = 8.5 ± 0.98; VAS lifting chair = 6.63 ± 1.21;VAS working = 6.38 ± 1.01; VAS nighttime = 7.04 ± 1.2;); 24 weeks posttherapy VAS resting = 3.67 ± 1.63; VAS stretching = 0.71 ± 1.83; VAS pressed = 5.88 ± 1.9; VAS lifting chair = 4.58 ± 1.61; VAS working = 4.63 ± 1.31; VAS nighttime = 3.38 ± 1.71)
[42]	Pettrone et al.	114	ESWT vs placebo	2000 (1)	0, 2, 4, 8, and 12 weeks	*Significant pain reduction (p = 0.001) and improvement of functional scores (p < 0.01) of the ESWT group compared to the placebo at 12 weeks after the therapy. * ESWT Pain (baseline = 74 ± 15.8; posttreatment = 37.6 ± 28.7); placebo pain 75.6 ± 16.0 51.3 ± 29.7 32% 0.02 ESWT Functional scale(baseline = 4.7 ± 1.8; posttreatment=2.3 ± 1.6); placebo Functional scale(baseline = 4.6 ± 1.8 posttreatment = 3.2 ± 2.1)
[43]	Trentini et al.	37	ESWT	1000 (4)	0 and last follow-up (Nd)	*No positivity to clinical assessment test (p < 0.01) for lateral epicondylitis after the treatment and betterment of outcome and pain (p < 0.001)*. quickDASH score (baseline = 51.6; posttreatment = 5.5) VAS (pain baseline = 8; posttreatment = 1.1)
[44]	Guller et al.	20	ESWT vs placebo	1500 (Nd)	0, End of treatment and 1 month	* Successful results in ESWT group, compared to the other, in pain and functional outcome, but no significant difference in the grasp and pinching strength between the measurements of the groups (p > 0.05).* ESWT VAS(baseline = 5.8 ± 1.8; posttreatment = 4.3 ± 2.1); placebo VAS(baseline = 6.1 ± 1.6;posttreatment=5.3 ± 1.8); ESWT PRTEE(baseline = 79.7 ± 26.4;posttreatment=60.1 ± 33.2); placebo PRTEE(baseline = 76.7 ± 19.7; posttreatment = 64.7 ± 20.2)
[45]	Radwan et al.	56	ESWT vs Tenotomy	100+1400 (Nd)	0, 3, 6, 12 weeks and 1 year	*Between the 3th and the 12th weeks, improvement of each assessment in both the treatments. Similar functional results (p > 0.05) were found between the ESWT and operative groups*. Success (Roles and Maudsley excellent and good results) = ESWT (3 weeks = 48.3%; 6 weeks = 58.6%; 12 weeks = 65.5%; 1 years = 62.10%); Tenotomy (3 weeks = 59.3%; 6 weeks = 63.0%; 12 weeks = 74.1%; 1 year = 77.80%)
[46]	Lee et al.	22	ESWT vs steroid local injection	2000 (1)	0, 1, 2, 4, 8 weeks	*Significant statistical differences were found between pre- and post-therapy in both the groups. Early better outcome was recorded in the local steroid injection group, but similar values were reported in the subsequently follow-ups.* 1-week follow up (ESWT Excellent-Good = 41.7%; steroid local injection Excellent-Good = 70.0%; ESWT Acceptable-Poor = 58.3%; steroid local injection Acceptable-Poor = 30.0%); 8-week follow up(ESWT Excellent-Good = 66.7%; steroid local injection Excellent-Good = 40.0%; ESWT Acceptable-Poor = 33.3%; steroid local injection Acceptable-Poor = 60.0%)
[47]	Vulpiani et al.	80	ESWT vs Cryoultrasound	2400 (2-3)	0, 3, 6, 12 months	*In the short term, the two treatments results were found similar in pain assessment and patients satisfaction rate. In middle and long-term better result in ESWT group than the other were found. * ESWT VAS (baseline = 6.52 ± 1.47; last follow-up = 2.32 ± 2.25) Cryo-US VAS(baseline = 6.60 ± 1.64; last follow-up = 4.70 ± 2.79)
[48]	Wong et al.	34	ESWT vs Acupuncture	2000 (Nd)	0, End of treatment and 2 weeks	*Similar results between two treatment groups in all the parameters. At the end of the therapies the no further pain improvements were recorded.* Acupunture VAS (baseline = 6.12 ± 2.09; 2weeks follow-up = 4.06 ± 2.41); Acupunture DASH (baseline = 66.88 ± 14.13; 2weeks follow-up = 63.94 ± 15.34); ESWT VAS (baseline = 5.47 ± 1.97; 2weeks follow-up = 3.18 ± 2.13); ESWT DASH( baseline = 64.65 ± 14.56; 2weeks follow-up = 60.12 ± 15.50);

ESWT = extracorporeal shockwave therapy; DS = dietary supplement; Mayo = Mayo Elbow Performance Score; VAS = Visual Analogue scale; Patient Rated Tennis Elbow Evaluation Test = PRTEE-T; NSAIDs = nonsteroidal anti-inflammatory drug.
